# Bat echolocation signals based on the time-varying autoregressive method

**DOI:** 10.1186/s12983-025-00573-3

**Published:** 2025-07-30

**Authors:** Xuan Zhong, Zhongbao Wang, Jianshu Wang, Kuiying Yin, Jinhong Luo

**Affiliations:** 1 Nanjing Research Institute of Electronics Technology, Nanjing, 210039 Jiangshu China; 2 National Key Laboratory of Radar Detection and Sensing, Nanjing, 210039 Jiangshu China; 3 Institute of Evolution and Ecology, School of Life Sciences, Central China Normal University, Wuhan, 430079 Hubei China

**Keywords:** Bat, Echolocation signal, Vocal system, System modeling, TV-AR

## Abstract

Bat echolocation is among the most efficient biological sonar known to human, and is highly valuable for biomimetic research. Most bats produce dynamically changing echolocation signals, which is the key to high task performance. Although considerable progress has been made in bat sonar bionics research, the working mechanism of the bat sonar system has not yet been fully revealed, mainly reflecting the imperfect parameterized model of the bat vocal system. This paper describe the bat echolocation signal production as a time-varying autoregressive (TV-AR) model, and the trajectory of model parameter changes is modeled as segmental constant and continuous change. Based on the two forms of parameter changes, this paper use the regularized least squares method and the basis function method for parameter solving, respectively. The TV-AR based system model realizes the simulation of bat vocal system with Gaussian white noise as input and bat echolocation signal as output. Using echolocation signals recorded from the Pratt’s roundleaf bats performing an approach-and-land task in the laboratory, we show that naturalistic echolocation signals can be simulated from the proposed TV-AR with high quality. Preliminary simulation and analysis suggests that the model can also be extended to simulate echolocation signals of distinct bat species.

## Introduction

The excellent echolocation ability of bats comes from their sonar system, which can provide rich target auditory clues for bats, such as target distance, relative velocity, volume size, azimuth, elevation, and target texture information [1–3]. Bats can analyze auditory cues of targets with a resolution of  10ns and complete complex biological behaviors of detecting targets, with efficiency, power consumption, and environmental adaptability that modern sensors cannot match [4]. In addition, bats adjust the parameters of the transmitted signal based on the received echo signals to optimize perception tasks. For example, many bats that feed on insects will continuously shorten the duration and interval of sound wave emission through the target search, target tracking, and final hunting stages, and change the spectral and beam width of sound wave emission to improve target detection and capture success rates [5–7]. The excellent echolocation perception ability of bats has attracted the attention of researchers in multiple fields such as biology, psychology, aviation, and electronic engineering. Researchers in the field of radar explore new multi harmonic radar systems by studying the multi harmonic characteristics of bat active sonar systems [8–11]. Researchers in the field of autonomous driving have applied bat active sonar systems to the three-dimensional positioning and navigation of vehicles [12]. In terms of psychoacoustics, the clue perception ability of bat sonar systems provides a solution for the auditory perception of visually impaired individuals [13–15]. Echolocation bionics is developing into a very important field, but there is still a lack of research on the deep biological perception mechanism of bat sonar systems, mainly reflected in the incomplete parameterization model of bat vocal systems.

Currently, research on modeling bat vocal systems can be divided into two categories based on content: numerical studies of bat radiated sound fields and biomimetic modeling studies of bat vocal systems. The former conducts numerical analysis on the acoustic field during bat echolocation, constructs a physical model of the external organs emitted or received by bats through finite element method, simulates the acoustic field after bats emit echolocation signals, and explores the acoustic functions of various external organs of bats. For example, Müller proposed a beam prediction method based on finite element method for bat emission and reception sound waves based on the results of tomography scans of bat nasal lobes and external ears, predicting the emission and reception beams of many bat species [16–18]. Wang et al. conducted a numerical study on the acoustic effects of the nasal lobe structure of different bats, including the Rhinolophus paradoxolophus, Myotis ricketti and woolly horseshoe bat, they found that the oscillation of the saddle shaped structure of the nasal lobe of Rhinolophus paradoxolophus affects the echolocation function, the sonar characteristics of obstacle avoidance in Myotis ricketti, and the acoustic function of the ear canal under the tragus in woolly horseshoe bat [19–22]. Researchers have also conducted biomimetic modeling and analysis of bat vocal systems, but such studies are relatively scarce. For example, Herman et al. used an Autoregressive Moving Average (ARMA) model to perform power spectrum analysis on the echolocation signals of the bat, attempting to establish a vocal system model, and discussed the application prospects of this model in bat species identification [23]. Li et al. and Ma et al. used tomography technology to construct a three-dimensional digital model of the vocal tract of horseshoe bats in order to study the filtering effect of the nasal cavity and tracheal chamber on glottic airflow [24, 25]. They obtained the sound field distribution of the nasal cavity and tracheal chamber, and then constructed a physical model of some vocal organs. The results showed that the nasal cavity can increase the sound pressure of the second harmonic, and the tracheal chamber can block the reflection of the second harmonic, which has a protective effect on internal vocal organs. The research on modeling bat vocal systems in the above work mainly focuses on the physical modeling of some vocal organs, lacking a comprehensive understanding of the vocal system, which affects the in-depth exploration of the operational logic of bat sonar systems. Further research is needed on the bat vocal system model, combined with vocalization mechanism and signal processing methods, to establish a more comprehensive bat vocal system model.

This paper explores the feasibility of a parameterized model for bat vocal systems based on the TV-AR model. We consider all vocal organs as a system entity and use the TV-AR model to describe it. The system input is Gaussian white noise and the output is bat echolocation signal. We propose two hypotheses regarding the trajectory of changes in model parameters, namely dividing the proposed bat vocal system model based on the TV-AR model into two sub models. These two sub models and their corresponding hypotheses are as follows:

- In sub model 1, we assume that during the vocalization process of bats, the degree of change in their various vocal organs varies at different time periods. The degree of change over a period of time is relatively small, and the parameters of the vocal system can be approximated as unchanged during this period. However, there are significant differences in the parameters between different time periods. That is, the parameters of the bat vocal system model are approximated as piecewise constants. This sub model is referred to as the piecewise constant bat vocal system model in this paper.
- In sub model 2, we assume that during the vocalization process of bats, their various vocal organs continue to function, and the parameters of the vocal system change continuously over time without any discontinuity points. This sub model is referred to as the coefficient continuous variation bat vocal system model in this paper.

For the proposed sub model 1, this paper uses a change point detection method based on Group-Lasso [26] to obtain the mutation time point sequence and corresponding change amplitude sequence of the vocal system model parameters. The corresponding segmented parameters are obtained through the change amplitude sequence, and the order of the model is selected based on the results of four order criteria [27]. For sub model 2, this paper selects multiple basis function sequences and uses the basis coefficient expansion method to solve the model parameters [28]. We consider the time-varying parameters of the vocal system as a linear combination of basis functions, and constrain them within a subspace formed by a set of time functions. In this subspace, the time-varying parameters can be represented by time invariant basis coefficients, thereby transforming the problem of solving time-varying parameters into a time invariant problem. The model order is selected based on time-varying optimal parameter search (TV-OPS) [29], and the basis function dimension is selected based on modeling error and error reduction rate. After obtaining the model parameters, we take Gaussian white noise as the system input, analyze the similarity between the system output signal and the bat echolocation signal, and take the similarity between the two as the matching degree between the proposed bat vocal system model and the bat vocal organs.

The structure of this paper is as follows: Sect. 2 introduces the equivalent model of the bat vocalization system established in this paper. Section 3 introduces the parameter solution of the bat vocalization system model. In Sect. 4, the feasibility and superiority of the vocal system model proposed in this paper is verified by the measured bat data under the matching degree criterion selected in this paper.

## Bat vocalization mechanism and system modeling

### Vocalization mechanism of bat

**Fig. 1:**
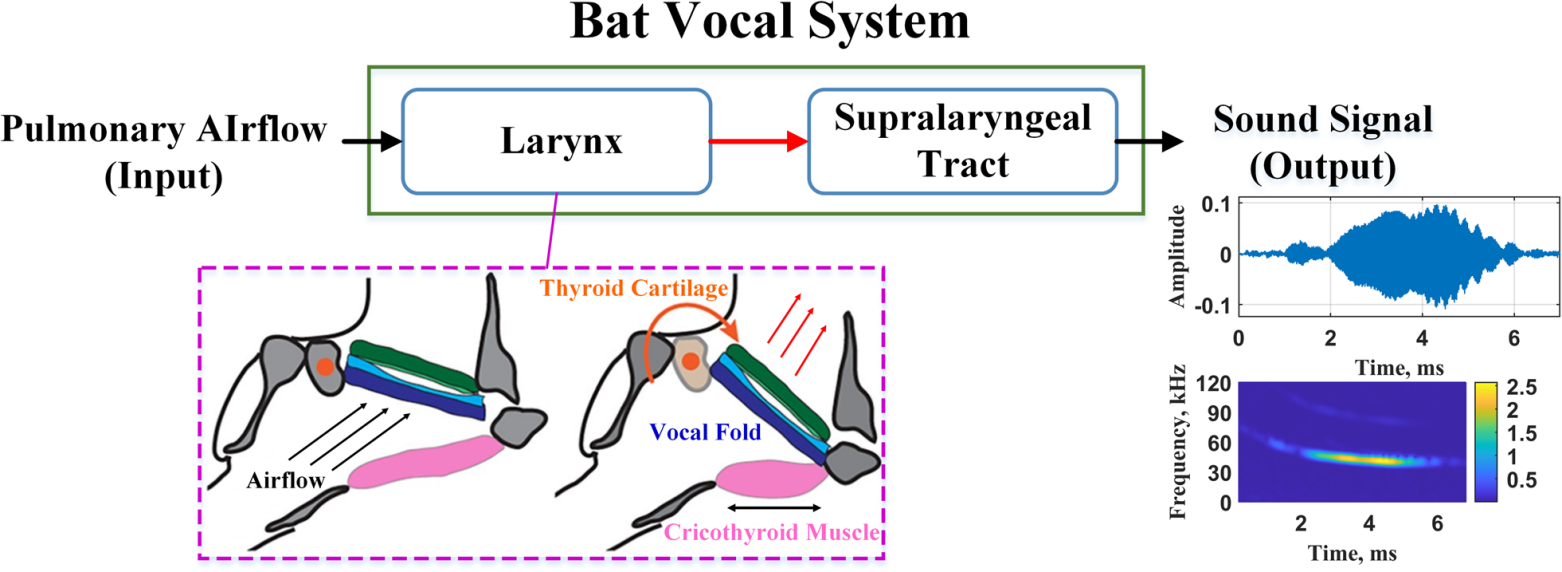
The process of bat vocalization. The schematic diagram of the larynx described in the purple dashed box is taken from this paper [32] and has been authorized

The vocal process of bats mainly consists of three parts: respiratory movement, laryngeal activity and supralaryngeal activity. When the bat exhales, the airflow from the lungs as input passes through the glottis of the larynx, which makes the vocal fold vibrate continuously. The tension of the vocal flod determines the basic frequency of the vibration, and completes the modulation of the frequency parameters of the fundamental frequency (and its harmonics) of the signal. The signal passes through the supralaryngeal of the bat (such as oral cavity and nasal cavity), and is emitted after the energy modulation of the frequency axis is completed by controlling the supralaryngeal strucutre [30, 31]. The tension of the vocal cord is not changed by the direct action of the vocal muscle, but by an indirect mechanism. Specifically, it is by contracting the cricothyroid muscle to tilt the thyroid cartilage inward, contract the vocal fold, and produce a higher vibration frequency. On the contrary, relax the cricothyroid muscle, relax the vocal cord, and reduce the vibration frequency. Figure 1 shows the process of bat vocalization.

### Modeling of bat vocal system

In this paper, the larynx and supralaryngeal of bats are regarded as a whole, which is called bat vocal system for short. This system takes the pulmonary airflow as the system input and the echolocation signal as the system output. In physics, the vocal system can be considered to be composed of multiple formants. Each formant resonates with a specific frequency band of the input signal, making the pulmonary airflow become an echolocation signal with a specific time-frequency structure and energy distribution under the joint action of multiple formants. This process is similar to the signal filtering process. The all pole model has a wide range of applicability, which can better describe the relationship between the input and output of the system and the physical meaning behind it. A single formant can be represented by a second-order all pole model, and the whole system can be composed of multiple second-order all pole models in cascade. ?The all-pole model is a system model characterized solely by resonances (without anti-resonances) [33]. The current signal value is primarily determined by a linear combination of its past values, augmented by the contribution of an excitation source. Its core concept relies on linear prediction, where the resonant characteristics of the signal are characterized by solving for the prediction coefficients. Considering the time-varying vocal organs of bats, this paper uses the system function of the time-varying all pole model to describe the bat vocal system. The system function $\text {H}\left( {n,z} \right)$ is obtained based on the Z-transform, and its expression is as follows:

1 $$\begin{aligned} \mathrm{{H}}\left( {n,z} \right) = \prod \limits _k^{p/2} {{\mathrm{{H}}_k}\left( {n,z} \right) } = \frac{1}{{1 + \sum _{i = 1}^p {{c_i}\left( n \right) {z^{ - i}}} }} \end{aligned}$$

where $n=0,1,\cdots ,N$ is the signal sampling time, *p* is the order of the model, $c_i(n)$ represents the coefficient of the *i*-th time-varying all pole model at time *n* and $z^{-i}$ represents the delay operator, corresponding to the *i*-step delay in the time domain.

It is difficult to obtain the exact signal form of the pulmonary airflow, so it can be generally considered as a random signal whose amplitude distribution obeys the standard Gaussian distribution, namely Gaussian white noise. When the input is Gaussian white noise, the time-varying all pole model is consistent with the TV-AR model [34]. The former describes the problem from the perspective of the system, while the latter describes the problem from the perspective of the signal. The parameters solved are also one-to-one corresponding. For example, the system poles can be obtained by finding the roots of the autoregressive coefficient polynomial. Therefore, this paper uses the term TV-AR model to describe the problem, that is, the bat vocal system is modeled as a TV-AR model. The echolocation signal of bats is recorded as *x*(*n*), and its vocal system can be expressed as follows:

2 $$\begin{aligned} x( n ) = \sum \limits _{i = 1}^p {{c_i}( n )x( n - i )} + \upsilon (n) \end{aligned}$$

where $\upsilon ( n ) \sim {\text {W}}{\text {N}}( 0,\sigma _\upsilon ^2 )$ is the Gaussian white noise input with mean value of 0 and variance of $\sigma _\upsilon ^2$.

**Fig. 2:**
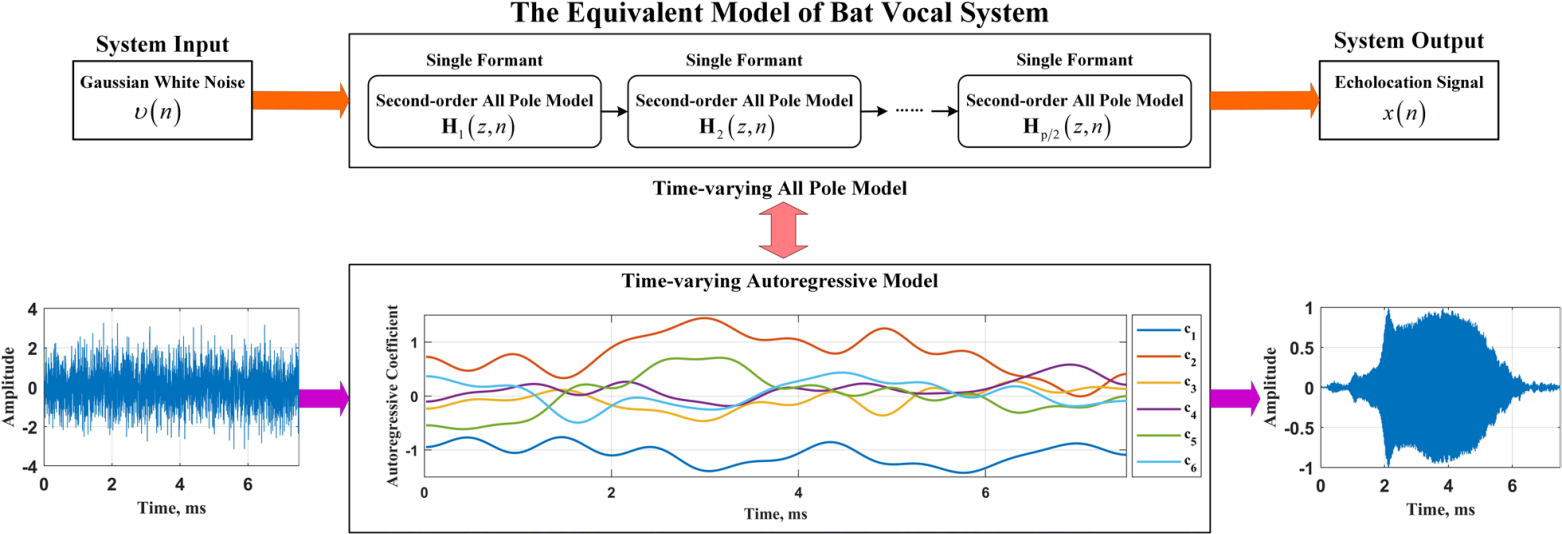
Equivalent model of bat vocal system based on TV-AR model, taking 6th order as an example

Figure 2 briefly shows the equivalent model of bat vocal system established in this paper, taking the 6th order model as an example.

Due to the lack of relevant research on bat vocal system model and the lack of prior information on the changes of its vocal system parameters with time, according to the characteristic that the output characteristics of the system with Gaussian white noise input are completely determined by the system parameters, this paper puts forward two conjectures on the change trajectory of the model parameters, namely, the parameters of piecewise constant and the parameters of continuous coefficient change. The TV-AR model used is piecewise constant autoregressive model and coefficient continuous variation autoregressive model, respectively.

## Parameter solution of bat vocal system model

### Parameter solution of sub model 1

#### TV-AR coefficient solution of sub model 1

As mentioned above, the TV-AR model used in sub model 1 is a piecewise constant coefficient autoregressive model, and the solution of the model parameters of the bat vocal system is transformed into the solution of the piecewise constant autoregressive coefficient. At present, there are many methods to solve the piecewise constant coefficient autoregressive model, which can be divided into two categories. The first category is to keep the autoregressive coefficient constant in the piecewise by regularized least square criterion [26, 35, 36], and the second category is to segment the signal by Bayesian method and Markov chain Monte Carlo methods (MCMC) [37, 38]. These methods can find the change point with high accuracy. In this paper, the first method is selected, and the autoregressive coefficient change point time is obtained by the change point detection method based on Group-Lasso, and its autoregressive coefficient is solved at the corresponding time.

Equation (2) describes a time-varying autoregressive model, which is now rewritten as follows:

3 $$\begin{aligned} x_n = \textbf{h}_n^{\text {T}}{\textbf{c}_n} + \upsilon _n \end{aligned}$$

where, subscript $n=0,1,\cdots ,N$ represents the sampling time, $\textbf{h}_n=\left[ x_{n-1},x_{n-2},\cdots ,x_{n-p}\right] ^{\text {T}}$ represents the autoregressive vector at time *n*, and $\textbf{c}_n=\left[ c_{1,n},c_{2,n},\cdots ,c_{p,n}\right] ^{\text {T}}$ represents the autoregressive coefficient vector at time *n*.

Since the autoregressive coefficient is assumed to be a piecewise constant, the autoregressive coefficient vector $\textbf{c}_n$ can be further written as:

4 $$\begin{aligned} \begin{array}{*{20}{c}} {{\mathbf{{c}}_n} = {{\textbf{c}}'_k},}&{{n_k} \le n \le {n_{k + 1}} - 1} \end{array} \end{aligned}$$

where ${\textbf{c}}'_k$ is a constant vector, $k=0,1,\cdots ,K$, *K* is the total number of change points, $n_k$ is the starting time of the x-th change point, and interval $\left[ n_k,n_{k+1}-1\right]$ is the existence period of the *k*-th segment signal.

For convenience, set the starting point $n_0$ of the first segment signal to 0, and the ending point $n_ {k+1}-1$ of the *K*-th segment signal to *N*. In this way, the problem can be described as estimating the time sequence $\{n_k\}_{k = 1}^K$ corresponding to the change point, in which the given signal sequence $\{x_n\}_{n = 0}^N$ is divided into $K+1$ segmented signals, and the corresponding autoregressive coefficient ${\textbf{c}}'_k$ is solved for each segmented signal sequence $\{ {{x_n}} \}_{n = {n_k}}^{{n_{k + 1}} - 1}$. This problem can be solved by regularized least square method, which is mathematically expressed as follows:

5 $$\begin{aligned} \{ {{{{{\hat{\textbf{c}}}}}_n}} \}_{n = 0}^N = \mathop {\arg \min }\limits _{\{ {{\mathbf{{c}}_n}}\}_{n = 0}^N} \left[ {\frac{1}{2}\sum \limits _{n = 0}^N {{{( {{x_n} - \mathbf{{h}}_n^{\text {T}}{\mathbf{{c}}_n}} )}^2} + \lambda \sum \limits _{n = 1}^N {\delta ( {{\mathbf{{c}}_n} - {\mathbf{{c}}_{n - 1}}} )} } } \right] \end{aligned}$$

where $\{ {{\hat{\textbf{c}}}}_n \}_{n = 0}^N$ represents the estimated value of the autoregressive coefficient $\{ \textbf{c}_n \}_{n = 0}^N$, $\lambda$ is an adjustment coefficient with a positive value, and $\delta (\cdot )$ represents the regularization function, also known as the penalty function.

The larger the value of $\lambda$, the greater the influence of the penalty function, and the fewer change points obtained. Conversely, the more change points obtained, the more change points there are. For example, if the adjustment coefficient is a very small positive number, even if there is only a slight change in the parameters between two sampling points, it will be judged as a change point. There will be a considerable number of change points throughout the entire signal duration, which is close to the coefficient variation trajectory of the autoregressive model with continuous coefficient variation.

In order to solve the Eq. (5), the equation needs to be transformed and expressed as a sparse regression problem with nonconvex regularization. The $\sum _{n = 0}^N {{{( x_n- \textbf{h}_n^{\text {T}}\textbf{c}_n)}^2}}$ part of Eq. (5) can be re expressed as:

6 $$\begin{aligned} \sum \limits _{n = 0}^N {{{( {{x_n} - \textbf{h}_n^{\text {T}}{{\textbf{c}}_n}})}^2}} = \Vert {{\textbf{y}} - \mathbf{{Mc}}} \Vert _2^2 \end{aligned}$$

where $\textbf{y}=\left[ x_0,x_1,\cdots ,x_N\right] ^{\text {T}}$ is the observation vector of the echolocation signal, and $\textbf{c}=\left[ \textbf{c}^{\text {T}}_0,\textbf{c}^{\text {T}}_1,\cdots ,\textbf{c}^{\text {T}}_N\right] ^{\text {T}}$ is the TV-AR coefficient expanded according to the time sampling points, $\textbf{M}=\left[ \textbf{m}_0,\textbf{m}_1,\cdots ,\textbf{m}_n,\cdots ,\textbf{m}_N\right] ^{\text {T}}$, $\textbf{m}_n=\left[ \mathbf{{0}}_{np}^{\text {T}},{\textbf{h}}_n^{\text {T}},\mathbf{{0}}_{(N-n)p}^{\text {T}} \right] ^{\text {T}}$, $\mathbf{{0}}_{L}$ represents all zero column vector with length *L*.

The entire sequence of change points is defined as $\textbf{d}=\left[ \textbf{d}_0^{\text {T}},\textbf{d}_1^{\text {T}},\cdots ,\textbf{d}_N^{\text {T}} \right] ^{\text {T}}$, where $\textbf{d}_n$ is a sequence vector of change points with a length of *p*, representing the difference of TV-AR coefficients of adjacent time sampling points, and its expression is as follows:

7 $$\begin{aligned} \textbf{d}_n =\left\{ {\begin{array}{*{20}{c}} {\textbf{c}_n}& {n = 0}\\ {{\textbf{c}_n} - \textbf{c}_{n-1}}& {others} \end{array}}\right. \end{aligned}$$

Write the above equation in matrix form:

8 $$\begin{aligned} \mathbf{{c}} = \left( {\mathbf{{T}} \otimes {\mathbf{{I}}_p}} \right) \mathbf{{d}} \end{aligned}$$

where ${\textbf{T}} \in {\mathbb {R}^{( {N + 1} ) \times ( {N + 1})}}$ is a lower triangular matrix with non-zero elements of 1, ${{\textbf{I}}_p} \in {\mathbb {R}^{p \times p}}$ is a unit matrix of order *p*, and $\otimes$ is the Kronecker product.

Combining Eqs. (6) and (8), Eq. (5) can be converted into the following form:

9 $$\begin{aligned} \{ {{{\hat{\textbf{d}}}}_n} \}_{n = 0}^N = \mathop {\arg \min }\limits _{\{ {{\textbf{d}_n}} \}_{n = 0}^N} \left[ {\frac{1}{2}\Vert {{\textbf{y}} - \mathbf{{Xd}}} \Vert _2^2 + \lambda \sum \limits _{n = 1}^N {\delta ( {{\textbf{d}_n}} )} } \right] \end{aligned}$$

where ${\textbf{X}} \in {\mathbb {R}^{( {N + 1} ) \times ( {N + 1})p}}$ is a matrix with the following structure:

10 $$\begin{aligned} \mathbf{{X}} = \left[ {\begin{array}{*{20}{c}} {\mathbf{{h}}_0^{\text {T}}}& {\mathbf{{0}}_p^{\text {T}}}& \cdots & \cdots & {\mathbf{{0}}_p^{\text {T}}}\\ {\mathbf{{h}}_1^{\text {T}}}& {\mathbf{{h}}_1^{\text {T}}}& {\mathbf{{0}}_p^{\text {T}}}& \cdots & {\mathbf{{0}}_p^{\text {T}}}\\ \vdots & \vdots & \vdots & \ddots & \vdots \\ {\mathbf{{h}}_{N - 1}^{\text {T}}}& {\mathbf{{h}}_{N - 1}^{\text {T}}}& \cdots & {\mathbf{{h}}_{N - 1}^{\text {T}}}& {\mathbf{{0}}_p^{\text {T}}}\\ {\mathbf{{h}}_N^{\text {T}}}& {\mathbf{{h}}_N^{\text {T}}}& \cdots & {\mathbf{{h}}_N^{\text {T}}}& {\mathbf{{h}}_N^{\text {T}}} \end{array}} \right] \end{aligned}$$

In this way, the solution object will change from the TV-AR coefficient sequence $\textbf{c}$ to the change point sequence $\textbf{d}$. Selecting the appropriate penalty function can make those relatively small vectors $\textbf{d}_n$ in $\textbf{d}$ become zero vectors, that is, the obtained change point estimation sequence ${{\hat{\textbf{d}}}}=\left[ \hat{\textbf{d}}_0^{\text {T}},{{\hat{\textbf{d}}}}_1^{\text {T}},\cdots ,{{\hat{\textbf{d}}}}_N^{\text {T}} \right] ^{\text {T}}$ is sparse between groups, in which the non-zero vector corresponds to the amplitude of the TV-AR coefficient $\textbf{c}$ mutation, which also corresponds to the properties of the piecewise constant coefficient autoregressive model. Group-Lasso can be seen as an extension of Lasso. Lasso selects the elements in the vector to make some elements become 0. Group-Lasso selects the vectors in the vector group to make some vectors become zero vectors. We apply Group-Lasso to Eq. (9), and the problem is transformed into estimating an inter group sparse vector:

11 $$\begin{aligned} \{ {{{\hat{\textbf{d}}}}_n} \}_{n = 0}^N = \mathop {\arg \min }\limits _{\{ {{\textbf{d}_n}} \}_{n = 0}^N} \left[ {\frac{1}{2}\Vert {{\textbf{y}} - \mathbf{{Xd}}} \Vert _2^2 + \lambda \sum \limits _{n = 1}^N { \Vert \textbf{d}_n \Vert _2^2} } \right] \end{aligned}$$

The upper bound $\lambda ^*$ of the adjustment coefficient $\lambda$ is related to the input signal $\textbf{y}$, and its relationship is as follows:

12 $$\begin{aligned} {\lambda ^ * } = {\max _{n = 1,...,N}}{\left\| {\mathbf{{X}}_n^{\mathrm{{T}}}\left( {{\mathbf{{X}}_{\mathrm{{0}}}}{\mathbf{{d}}_{0,c}} - \mathbf{{y}}} \right) } \right\| _2} \end{aligned}$$

where ${\textbf{X}_n} \in {\mathbb {R}^{N + 1 \times L}},n = 0,1,...,N$ is a submatrix of ${\textbf{X}} = \left[ {{{\textbf{X}}_0},{{\textbf{X}}_1},...,{{\textbf{X}}_N}} \right]$, ${{\textbf{d}}_{0,c}} = {\left( {{\textbf{X}}_0^{\text {T}}{{\textbf{X}}_0}} \right) ^{ - 1}}{\textbf{X}}_0^{\text {T}}{\textbf{y}}$, according to the suggestion in reference [26], the adjustment coefficient is set to 5-$20\%$ of $\lambda ^*$.

In short, when $\lambda = {\lambda ^ * }$, it means that under this threshold, the autoregressive coefficient has not changed during the signal duration, and the segmented constant autoregressive model degenerates into a constant autoregressive model. According to the adjustment coefficient setting suggestion [26], the adjustment coefficient $\lambda$ is set to 5-$20\%$ of $\lambda ^*$.

For Eq. (11), there are many methods to solve it. In this paper, the block coordinate descent method is used to solve it [26]. Limited space, the block coordinate descent method is not introduced here. After obtaining the sequence of change points $\{ {{{\hat{\textbf{d}}}}_n} \}_{n = 0}^N$, the TV-AR coefficient $\{ {{{\hat{\textbf{c}}}}_n} \}_{n = 0}^N$ can be obtained as follows:

13 $$\begin{aligned} {{\hat{\textbf{c}}}}_n = \sum \limits _{n'=0}^n {{{\hat{\textbf{d}}}}_{n'}} \end{aligned}$$

In addition, it should be pointed out that the above process is based on the assumption that the total number of change points is unknown in advance, which is also a common situation encountered in actual signal processing. Therefore, there is no expression for the interval length between adjacent change points. During the simulation process, the change points and their corresponding autoregressive parameters are solved using the block coordinate descent method, which is an iterative process. In the last iteration, the interval length of each change point can be obtained by outputting the corresponding sampling time along with the change points.

#### Order selection of sub model 1

At present, there are few order criteria for TV-AR model. One of the more applicable criteria is TV-OPS, this method is proposed for TV-AR model with base coefficient expansion, and is not suitable for piecewise constant coefficient autoregressive model [29]. Therefore, for sub model 1, we selected four widely applicable autoregressive model criteria: minimum mean square error criterion (MMSE), final prediction error (FPE) [39], Akaike Information Criterion (AIC) [40] and minimum description length (MDL) [41]. The latter three criteria monitor modeling errors through penalty functions, selecting the optimal model order based on error values to achieve a stable state in modeling error at that order.? However, in practical application, these four criteria can not give an exact minimum order, but only have certain reference significance. Therefore, based on these four criteria, this paper selects the order according to the decline rate of modeling error. When the decline rate of modeling error is lower than a certain threshold, select the corresponding order. The decline rate of modeling error ${v_\xi }\left( p\right)$ is defined as follows:

14 $$\begin{aligned} {v_\xi }\left( p\right) ={\left( \xi _p - \xi _{p-1} \right) ^2}/{\left( \xi _p \right) ^2} \end{aligned}$$

where $\xi _p$ is the modeling error of order *p*.

### Parameter solution of sub model 2

#### TV-AR coefficient solution of sub model 2

Sub model 2 assumes that the vocal organs of bats continue to function during the vocal process, and the corresponding vocal system parameters change continuously with time, and there is no mutation point. Based on this assumption, this paper expands the basis coefficient of TV-AR model. By selecting the appropriate basis function and basis function dimension, the TV-AR coefficient is constrained in a subspace formed by a set of time functions. In the subspace, the TV-AR coefficient can be expressed by time invariant basis coefficient, and the time-varying problem is simplified and transformed into a time invariant problem. The TV-AR coefficient $c_i(n)$ in Eq. (2) can be defined as follows:

15 $$\begin{aligned} c_i\left( n \right) = \sum \limits _{j = 0}^q {{c_{ij}}{f_j}\left( n \right) } \end{aligned}$$

where $\left\{ {{f_j}\left( n \right) ,0 \le j \le q} \right\}$ is a set of linearly independent basis functions defined on the time sampling interval $\left[ 1,2, \cdots ,N \right]$, *q* is the number of basis functions, also known as the dimension of the basis function, $\{c_{ij}\}$ is a set of basis coefficients, and $f_0(n)$ is usually set to 1 to represent the stationary part of the TV-AR coefficient.

In this paper, $\{ c_{ij} \}$ is solved by minimizing the mean square error. The expression of mean square error is as follows:

16 $$\begin{aligned} \xi = \sum \limits _{n = p + 1}^N {{\varepsilon ^2}\left( n \right) } = \sum \limits _{n = p + 1}^N {{{\left( {x\left( n \right) - \sum \limits _{i = 1}^p {\sum \limits _{j = 0}^q {{c_{ij}}{f_j}x\left( {n - i} \right) } } } \right) }^2}} \end{aligned}$$

From Eq. (16), it can be seen that the mean square error $\xi$ is a quadratic function of the base coefficient $\{ c_{ij} \}$. Finding the base coefficient corresponding to the first derivative of the mean square error $\xi$ as 0 can obtain the unique optimal estimate of the base coefficient under the criterion of minimizing the mean square error. The first derivative of the mean square error is as follows:

17 $$\begin{aligned} \begin{array}{*{20}{l}} {\frac{{\partial \xi }}{{\partial {c_{kl}}}}}& { = 2\sum \limits _{n = p + 1}^N {\varepsilon \left( n \right) \frac{{\partial \varepsilon \left( n \right) }}{{\partial {c_{kl}}}}} }\\ {}& { = 2\sum \limits _{n = p + 1}^N {\left( {x\left( n \right) - \sum \limits _{i = 1}^p {\sum \limits _{j = 0}^q {{c_{ij}}{f_j}\left( n \right) x\left( {n - i} \right) } } } \right) {f_l}\left( n \right) x\left( {n - k} \right) } } \end{array} \end{aligned}$$

where, $$1 \le k \le p$$, $$0 \le l \le q$$.

To solve $c_{ij}$, a cross-correlation function is defined:

18 $$\begin{aligned} {\phi _{ij}}\left( {k,j} \right) = \sum \limits _{n = p + 1}^N {{f_j}(n)}{f_l}\left( n \right) x\left( {n - i} \right) x\left( {n - k} \right) \end{aligned}$$

According to the property of cross-correlation function, ${\phi _{lj}}\left( {k,i} \right) = {\phi _{jl}}\left( {k,i} \right) = {\phi _{lj}}\left( {i,k} \right) = {\phi _{jl}}\left( {i,k} \right)$, let Eq. (17) be equal to 0, rearrange the summation order, and obtain the following equation:

19 $$\begin{aligned} \sum \limits _{i = 1}^p {\sum \limits _{j = 0}^q {{c_{ij}}{\phi _{lj}}\left( {k,i} \right) } } = {\phi _{l0}}\left( {k,0} \right) \end{aligned}$$

There are $p(q+1)$ base coefficients in total, which means that the system of linear equations composed of $p(q+1)$ in the form of Eq. (19) needs to be solved. First, write the base coefficients in vector form:

20 $$\begin{aligned} \textbf{c}= \left[ \textbf{c}_1,\textbf{c}_2, \cdots ,\textbf{c}_i, \cdots ,\textbf{c}_p \right] ^{\text {T}} \end{aligned}$$

where $\textbf{c}_i = \left[ {{c_{i0}},{c_{i1}}, \cdots ,{c_{iq}}} \right]$ is the base coefficient vector corresponding to the *i*-th autoregressive coefficient.

Secondly, the corresponding cross-correlation vector expression is as follows:

21 $$\begin{aligned} \textbf{r}= \left[ {{\mathbf{{r}}_1},{\mathbf{{r}}_2}, \cdots ,{\mathbf{{r}}_k}, \cdots ,{\mathbf{{r}}_p}} \right] ^{\text {T}} \end{aligned}$$

where, $${\mathbf{{r}}_k} = \left[ {{\phi _{00}}\left( {k,0} \right) ,{\phi _{20}}\left( {k,0} \right) , \cdots ,{\phi _{q0}}\left( {k,0} \right) } \right]$$.

Then a covariance matrix is defined according to the sequence of elements in $\textbf{c}$:

22 $$\begin{aligned} {\varvec{\Phi }_{ki}} = \left[ {\begin{array}{*{20}{c}} {{\phi _{00}}\left( {k,i} \right) }& \cdots & {{\phi _{0q}}\left( {k,i} \right) }\\ \vdots & \ddots & \vdots \\ {{\phi _{q0}}\left( {k,i} \right) }& \cdots & {{\phi _{qq}}\left( {k,i} \right) } \end{array}} \right] \end{aligned}$$

Use this covariance matrix $\varvec{\Phi }_{ki}$ to build a block covariance matrix, and the expression is as follows:

23 $$\begin{aligned} \mathbf{{R}} = \left[ {\begin{array}{*{20}{c}} {{\varvec{\Phi }_{11}}}& \cdots & {{\varvec{\Phi }_{1p}}}\\ \vdots & \ddots & \vdots \\ {{\varvec{\Phi }_{p1}}}& \cdots & {{\varvec{\Phi }_{pp}}} \end{array}} \right] \end{aligned}$$

In this way, Eq. (19) can be written in matrix form:

24 $$\begin{aligned} \textbf{R}\textbf{c}=\textbf{r} \end{aligned}$$

The base coefficient $\textbf{c}=\textbf{R}^{-1}\textbf{r}$ corresponding to each autoregressive coefficient can be obtained.

#### Selection of basis function and dimension

Aiming at the hypothesis that the parameters of the vocal system of sub model 2 change continuously with time, this paper uses three kinds of basis functions, namely, Legendre polynomial basis function, Chebyshev basis function and discrete cosine basis function, which have strong description ability and track the change trajectory of a large range accurately. For the convenience of expression, they are recorded as ${f_{lj}}(n)$, where $l = 1,2,3$. They are briefly introduced below:

1. 25 $$\begin{aligned} {f_{1j} }\left( n \right) = \frac{{\left( {2j - 1} \right) {f_1}\left( n \right) {f_{j - 1}}\left( n \right) - \left( {j - 1} \right) {f_{j - 2}}\left( n \right) }}{j} \end{aligned}$$
  where $$j \ge 2$$, $${f_0}\left( n \right) = 1$$, $${f_1}\left( n \right) = \frac{{2\left( {n - 1} \right) }}{{N - 1}} - 1$$.
2. 26 $$\begin{aligned} {f_{2j} }\left( n \right) = \cos \left[ {j{{\cos }^{ - 1}}\left( {\frac{{2\left( {n - 1} \right) }}{{N - 1}} - 1} \right) } \right] \end{aligned}$$
  (3) Discrete cosine basis function:
  27 $$\begin{aligned} {f_{3j} }\left( n \right) = \alpha \left( j \right) \cos \left( {\frac{{\pi j\left( {2n + 1} \right) }}{{2N}}} \right) \end{aligned}$$
  where $\alpha \left( j \right)$ is a scaling factor, and $\alpha \left( j \right) = 1$ is selected in this paper.

**Fig. 3:**
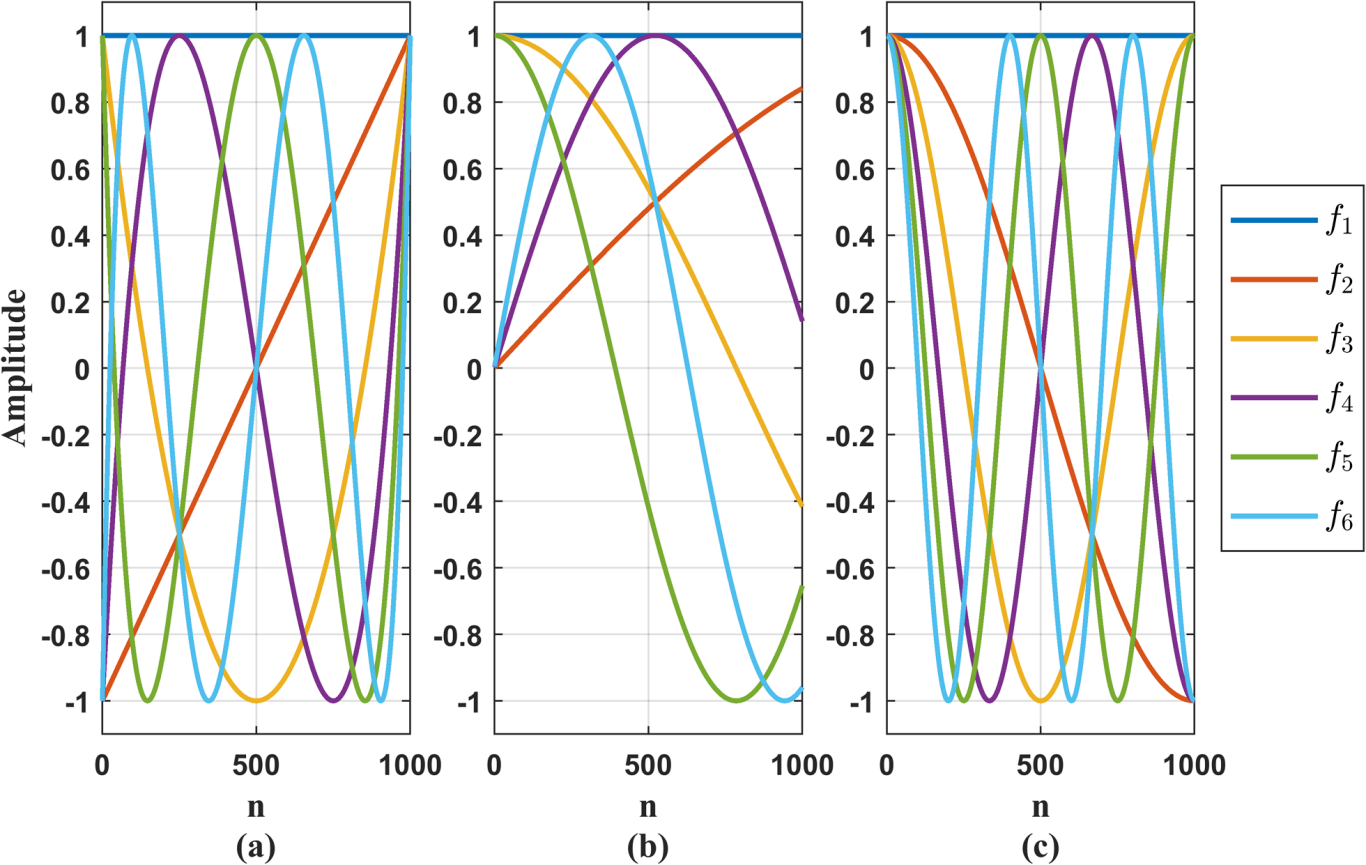
Three kinds of 6-dimensional basis functions. **a** 6-dimensional Legendre polynomial basis function; **b** 6-dimensional Chebyshev basis function; **c** 6-dimensional discrete cosine basis function

Figure 3 shows the time domain diagram of the above three basis functions in 6-dimensions.

At present, there is no clear criterion for the selection of the dimension of the basis function. In this paper, the dimension of the basis function is determined by the following methods:

Step 1 Select a TV-AR model order *p* and a basis function, and preset the falling speed threshold of modeling error $v_{\text {th}}$;
Step 2 Calculate the modeling error $\xi _p(j)$ and the decline rate $v_{\xi }(j)$ of the modeling error under different dimensions *j* of the basis function;
Step 3 Select the dimension ${\hat{j}}$ corresponding to the minimum value of modeling error; if the $\xi _p(j)$ continues to decrease with the increase of dimension *j*, proceed to step 4;
Step 4 Select the dimension ${\hat{j}}$ corresponding to the falling speed threshold $v_{\text {th}}$ of modeling error.

In the above steps, the decline rate $v_{\xi }(j)$ of modeling error with the dimension of the basis function is defined as follows:

28 $$\begin{aligned} {v_\xi (j)}={\left( \xi _p(j) - \xi _p(j-1) \right) ^2}/{\xi _p(j)^2} \end{aligned}$$

#### Order selection of sub model 2

For sub model 2, this paper selects TV-OPS to select the model order. Before selecting the order, we define several variable names. First, substitute Eq. (15) into Eq. (2):

29 $$\begin{aligned} x( n ) = \sum \limits _{i = 1}^p {\sum \limits _{j = 0}^q {{c_{ij}}{f_j}( n )x( n - i )} } + \upsilon ( n ) \end{aligned}$$

Then write Eq. (29) in matrix form:

30 $$\begin{aligned} \textbf{x}= {\textbf{W}_{\text {h}}}{\varvec{\theta }} + \varvec{\upsilon } \end{aligned}$$

where $\textbf{x}= {\left[ {x\left( 1 \right) ,x\left( 2 \right) , \cdots ,x\left( N \right) } \right] ^{\text {T}}}$, ${{\textbf{W}}_{\text {h}}} = \left[ {{\mathbf{{x}}_{10}}, \cdots ,{\mathbf{{x}}_{1q}}, \cdots ,{\mathbf{{x}}_{p0}}, \cdots ,{\mathbf{{x}}_{pq}}} \right]$ is the input matrix, and its column vector ${\textbf{x}}_{ij} = {\left[ {{f_j}\left( 1 \right) x\left( {1 - i} \right) , \cdots ,{f_j}\left( N \right) x\left( {N - i} \right) } \right] ^{\text {T}}}$ is the candidate vector, $\textbf{W}_{\text {h}}$ is the candidate vector pool, and $\varvec{\upsilon }$ is the Gaussian white noise vector.

Order selection using TV-OPS can be divided into four steps:

Step 1 Select a maximum linearly independent group from the candidate vector pool $\textbf{W}_{\text {h}}$, and record it as $\mathbf{{W}} = \left[ \textbf{w}_1,\textbf{w}_2, \cdots ,\textbf{w}_R \right]$, where ${\textbf{w}_k = [ w_{k1},w_{k2},\cdots ,w_{kN} ]^{\text {T}}}$;
Step 2 Replace $\textbf{W}_{\text {h}}$ in Eq. (30) with $\textbf{W}$, and find the least square solution of autoregressive coefficient $\varvec{\theta }$, which is recorded as ${\hat{\varvec{\theta }}} = \left[ {{\mathbf{{a}}_1},{\mathbf{{a}}_2}, \cdots ,{\mathbf{{a}}_R}} \right]$, where $\textbf{a}_k = [ {a_k}\left( 1 \right) ,{a_k}\left( 2 \right) , \cdots ,{a_k}\left( N \right) ]^{\text {T}}$;
Step 3 Calculate each vector in the vector group $\textbf{W}$ and its corresponding autoregressive coefficient vector as follows:
  31 $$\begin{aligned} {C_m} = \frac{1}{N}\sum \limits _{n = 1}^N {\sum \limits _{k = 0}^q {a_k^2\left( n \right) w_{m,k}^2\left( n \right) } } \end{aligned}$$
  where $${w_{m,k}} = {f_k}\left( n \right) x\left( {n - m} \right)$$.
Step 4 Discard the order corresponding to ${C_m}=0$ to complete the order selection.

## Experimental verification

**Fig. 4:**
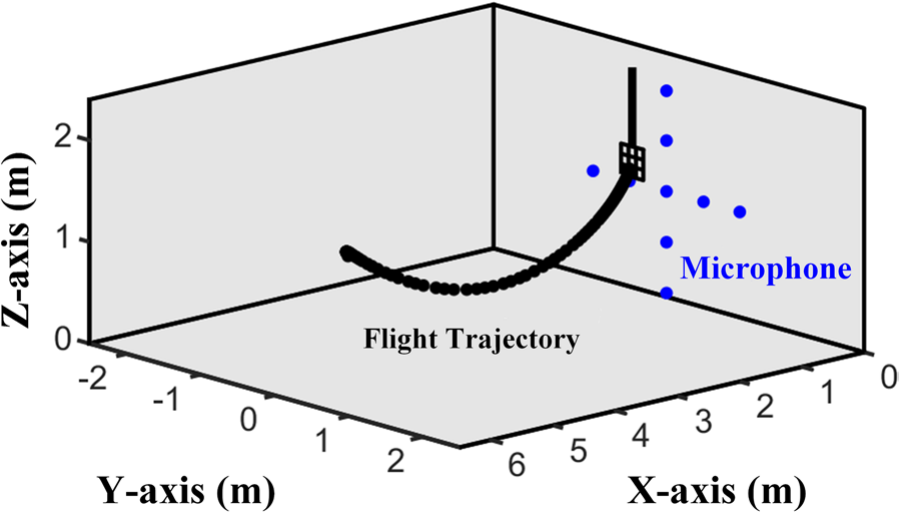
Echolocation signal recording environment of bats trained to approach and land on a perch

To validate the TV-AR model-based bat vocal system, we used natural echolocation signals of four adult Hipposideros pratti, two males and two females, engaged in a landing task [42]. Signals are recorded in a large room ($6.5\times 5\times 2.3$ m, length $\times$ width $\times$ height), the walls and ceiling are covered with 8 cm thick acoustic foam, and the floor is covered with nylon blanket to reduce echoes and reverberations. The landing perch ($20 \times 20$ cm) is suspended about 0.9 m from the ceiling and about 0.75 m in front of the microphone array. All four Hipposideros pratti were well trained and were able to fly approximately 4.5 meters along a consistent path from one side of the room to the landing perch on the other side of the room. Under quiet (noise free) conditions, 502 signals were recorded with a signal sampling rate of 192 kHz. This paper does not involve animal experiments, only the analysis and modeling of signal. The detailed process of bat training and echolocation signal data acquisition has been described in previous article [42]. Figure 4 shows the signal recording environment and one representative flight trajectory of a bat during a landing task.

### Sub model 1

**Fig. 5:**
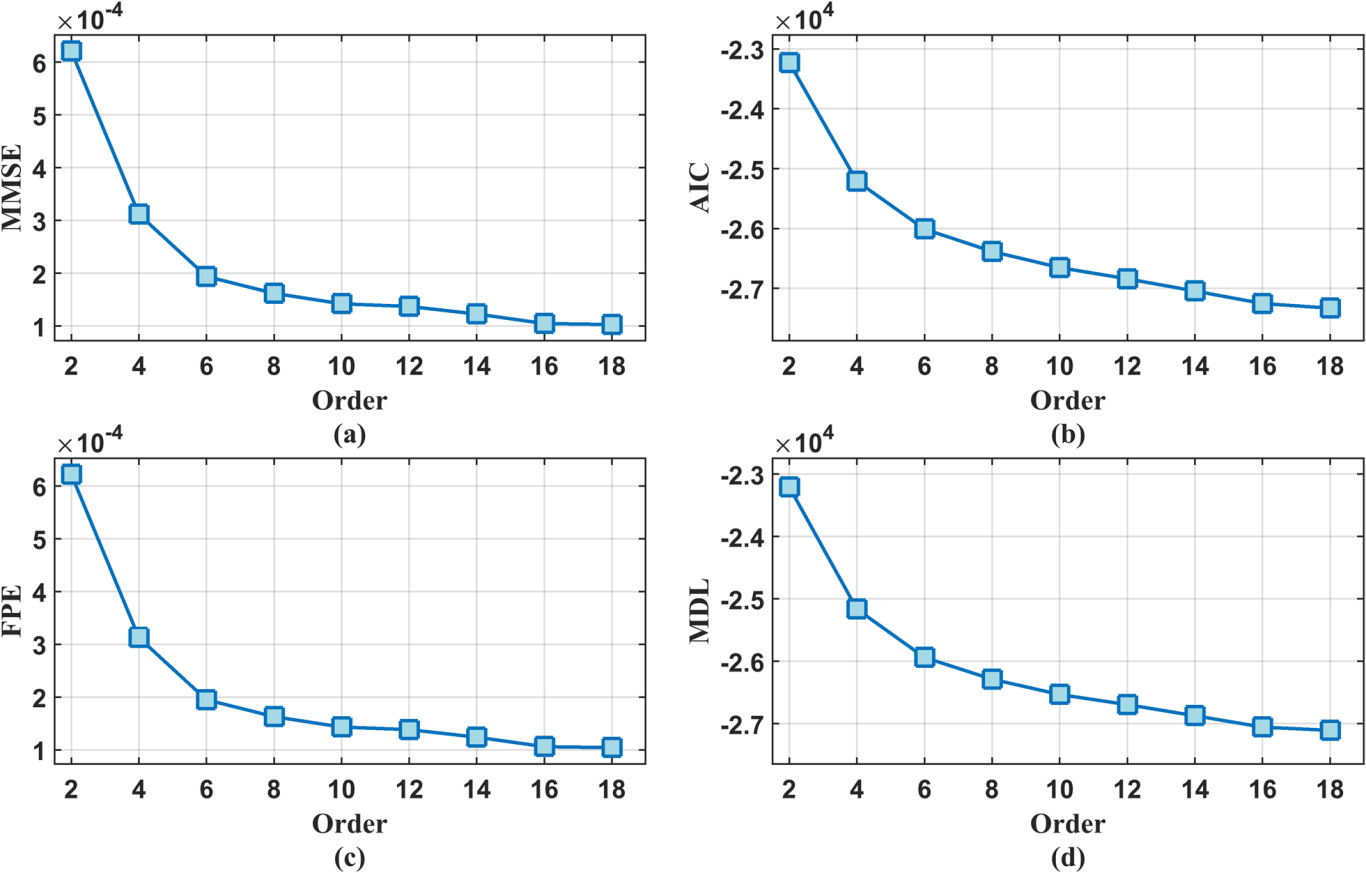
Selection results of four model order criterion. **a** MMSE criterion; **b** AIC criterion; **c** FPE criterion; **d** MDL criterion

To determine the optimal model order while avoiding overparameterization that compromises physical interpretability?, the maximum model order was constrained to 18. The piecewise autoregressive constant coefficients under each candidate order were computed to establish a piecewise-constant bat vocalization system?. Then calculate order selection results of MMSE criterion, AIC criterion, FPE criterion and MDL criterion under the corresponding order. Figure 5 shows the mean value of the four order selection results of 502 signals collected in this paper.

**Fig. 6:**
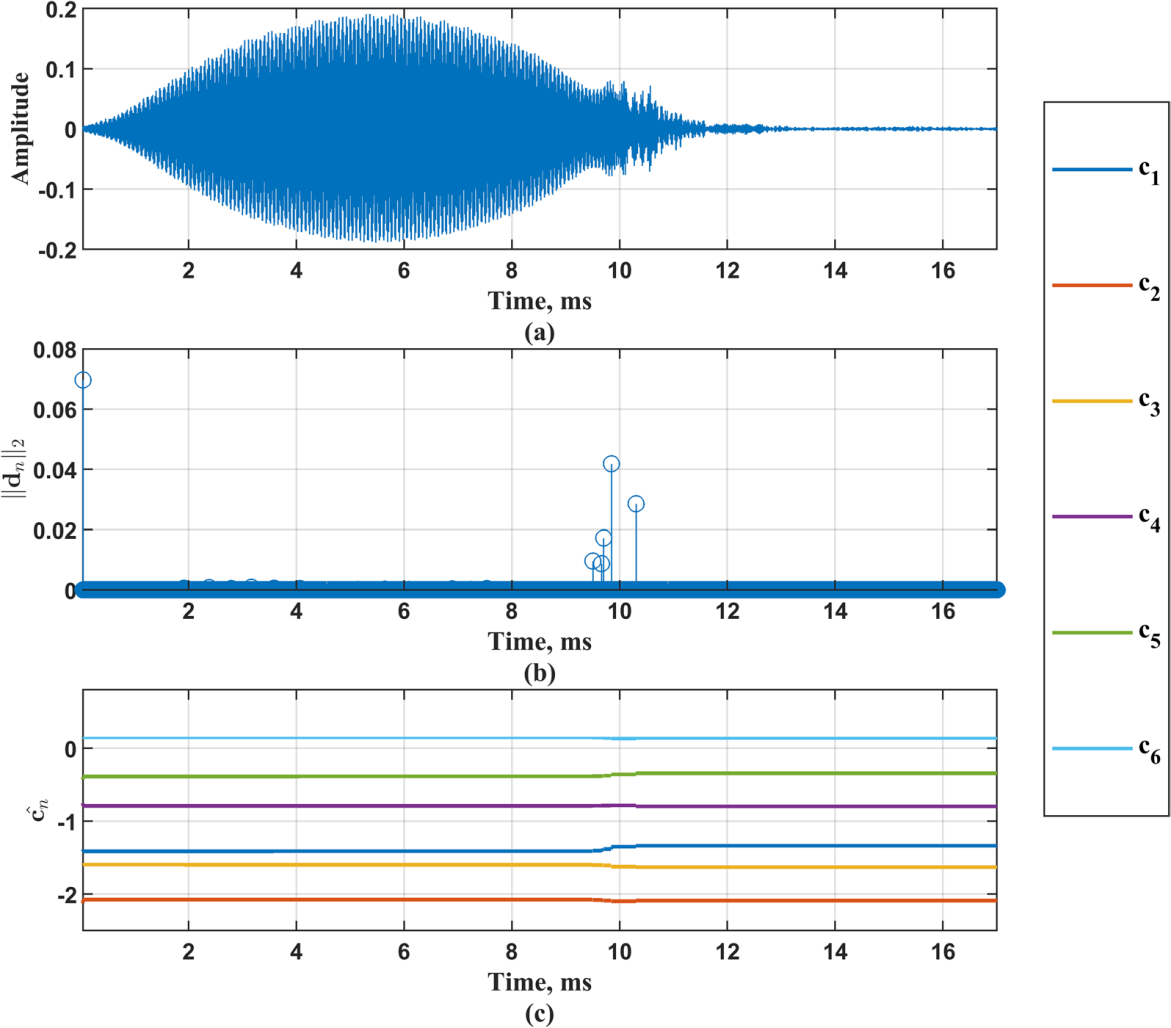
Change point sequence $\Vert \textbf{d}_n \Vert _2$ and piecewise autoregressive constant coefficient ${{\hat{\textbf{c}}}}_n$, and original signal time-domain diagram under 6-*th* order. **a** Time domain diagram of original signal; **b** Change point sequence $\Vert \textbf{d}_n \Vert _2$; **c** Piecewise autoregressive constant coefficient ${{\hat{\textbf{c}}}}_n.$

**Fig. 7:**
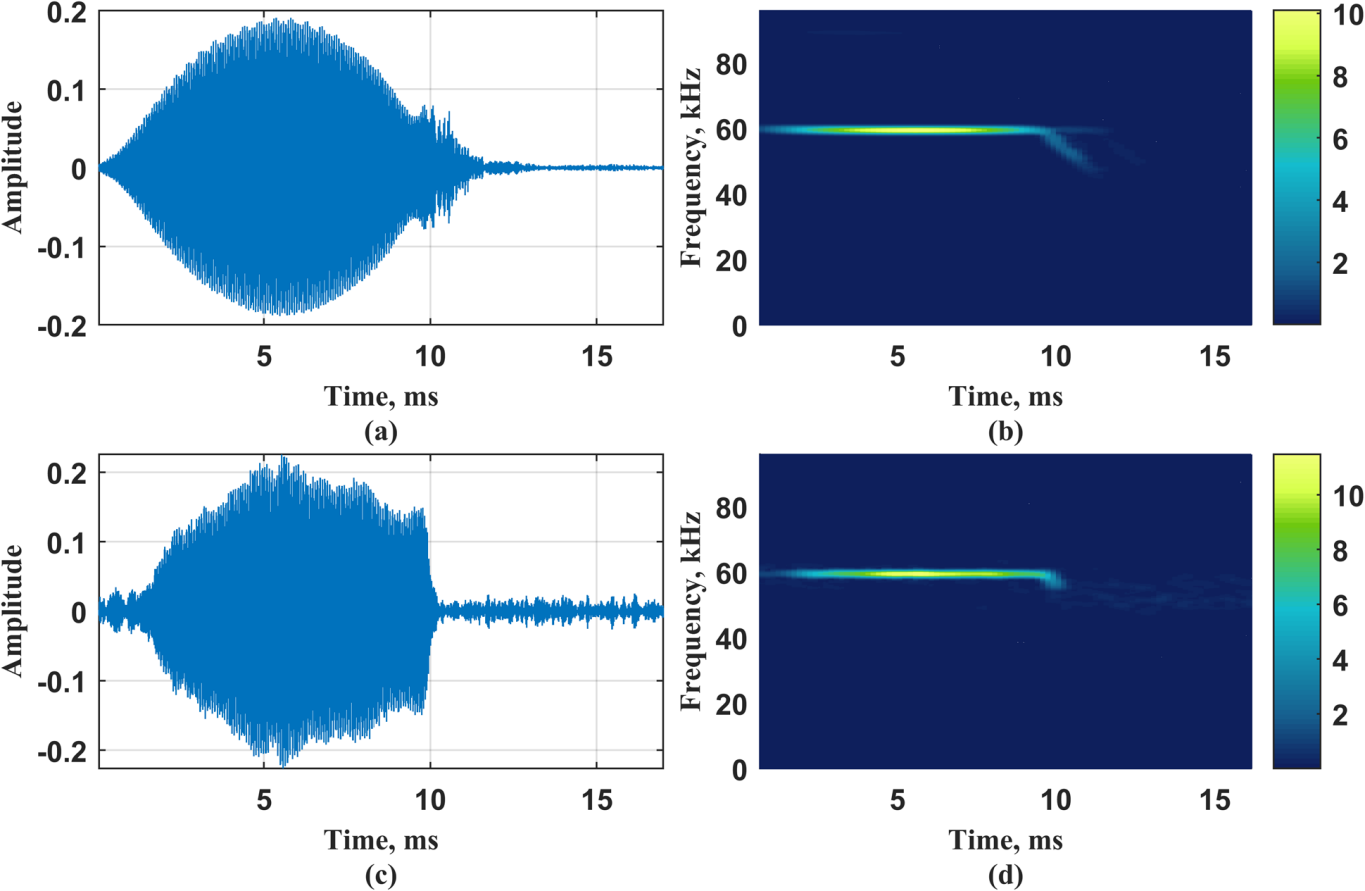
Output signal and original signal. **a** Time domain diagram of original signal; **b** Original signal STFT results; **c** Time domain diagram of output signal; **d** Output signal STFT results

As evidenced by the figure, the four order-selection criteria fail to converge to a definitive value with increasing model complexity?. To resolve this ambiguity while preserving physical interpretability against excessive parameterization?, we systematically evaluated five candidate orders (4-*th*, 6-*th*, 8-*th*, 10-*th*, 12-*th*) in subsequent analyses?. After the order of the model is selected, Eq. (9) is solved, where the adjustment coefficients are set as $5\%$, $10\%$, $15\%$ and $20\%$ of $\lambda ^{*}$ respectively. The sequence of change points obtained under different adjustment coefficients is calculated as Eq. (12), so as to obtain the corresponding piecewise autoregressive constant coefficient, and then construct the vocal system. The vocal system takes Gaussian white noise as the input and records the output signal as ${{\hat{\textbf{x}}}}_{\text {G}\text {L}}$, where subscripts $_{\mathrm{{GL}}}$ correspond to Group-Lasso. Figure 6 shows the change point sequence $\Vert \textbf{d}_n \Vert _2$, piecewise autoregressive constant coefficient ${{\hat{\textbf{c}}}}_n$, and original signal time-domain diagram of the bat vocal system model when the model order is 6-*th* and adjustment coefficients $\lambda$ is $15\%*\lambda ^{*}$. Figure 7 shows the time domain diagram of a certain original signal and its output signal, as well as the corresponding Short-Time Fourier Transform (STFT) results.

To assess the acoustic performance of sub model 1, the mean and variance of linear correlation between the original echolocation signals and output signals are adopted as evaluation criteria?. Given that the input signal constitutes a single realization of Gaussian white noise in practical applications, output variability necessitates conducting 5000 Monte Carlo trials?. Each trial computes the Pearson correlation coefficient between individual output signals and the original signal, with the mean of these coefficients serving as the linear correlation metric?. The linear correlation metrics corresponding to bat vocal system models is denoted as ${\rho _{\text {G}\text {L}}}\left( {j,k} \right)$, and its mathematical expression is as follows:

32 $$\begin{aligned} \rho _{\text {G}\text {L}}( {j,k} ) = \frac{1}{{{N_m}}}\sum \limits _{m = 1}^{{N_m}}{\frac{{Cov\left( {{{\textbf{x}}_k},{{{\hat{\textbf{x}}}}^m}_{\text {G}\text {L},j}} \right) }}{{{\sigma _{\textbf{x}}}{\sigma _{{{{\hat{\textbf{x}}}}^m}_{\text {G}\text {L},j}}}}}} \end{aligned}$$

where $N_m$ is the number of experiments, $\textbf{x}_k$ is the *k*-*th* echolocation signal, ${{\hat{\textbf{x}}}}_{\text {G}\text {L},j}^m$ is the output signal obtained from the *m*-*th* Monte Carlo test when the model order is *j*.

Calculate the linear correlation of all signals according to Eq. (32), and take the mean and standard deviation. The mean is recorded as $E_{\text {G}\text {L},j}$, and the standard deviation is recorded as $D_{\text {G}\text {L},j}$. The corresponding mathematical expression is as follows:

33 $$\begin{aligned} & {E_{\text {G}\text {L},j}} = \frac{1}{{{N_e}}}\sum \limits _k^{{N_e}} {{\rho _{\text {G}\text {L}}}\left( {j,k} \right) } \end{aligned}$$

34 $$\begin{aligned} & {{D}_{\text {G}\text {L},j}} = \sqrt{ \frac{1}{{{N_e}}}\sum \limits _{k = 1}^{{N_e}} {{{\left[ {{\rho _{\text {G}\text {L}}}\left( {j,k} \right) - {E_{\text {G}\text {L},j}}} \right] }^2}} } \end{aligned}$$

where $N_e$ is the number of signals.

Table 1 shows the mean and standard deviation of the corresponding linear correlation of the bat vocal system model based on the signals collected in this paper under four adjustment coefficients and five model orders.

**Table 1:** ${E}_{\text {G}\text {L},j}$ and ${D}_{\text {G}\text {L},j}$ of bat vocal system model based on the signals collected in this paper under four adjustment coefficients and five orders

Model Order(xx$\%*\lambda ^{*}$)	$\textbf{4}$-$\textbf{th}$	$\textbf{6}$-$\textbf{th}$	$\textbf{8}$-$\textbf{th}$	$\textbf{10}$-$\textbf{th}$	$\textbf{12}$-$\textbf{th}$
$E_{\text {G}\text {L},j}/{D}_{\text {G}\text {L},j}\left( {5\% } \right)$	0.472/0.012	0.537/0.012	0.582/0.012	0.594/0.012	0.607/0.012
$E_{\text {G}\text {L},j}/{D}_{\text {G}\text {L},j}\left( {10\% } \right)$	0.452/0.011	0.515/0.012	0.555/0.012	0.568/0.012	0.579/0.012
$E_{\text {G}\text {L},j}/{D}_{\text {G}\text {L},j}\left( {15\% } \right)$	0.437/0.011	0.496/0.012	0.530/0.012	0.545/0.012	0.558/0.012
$E_{\text {G}\text {L},j}/{D}_{\text {G}\text {L},j}\left( {20\% } \right)$	0.421/0.011	0.479/0.012	0.511/0.012	0.525/0.012	0.541/0.012

### Sub model 2

Similar to the verification process of sub model 1, first determine the order of the model, which is set to 4-*th*, 6-*th*, 8-*th*, 10-*th* and 12-*th* orders respectively. Under these five orders, the dimension of the basis function is determined by the basis function selection criteria described above. Secondly, verify the rationality of the order of the model through TV-OPS, then solve the TV-AR coefficient, take the Gaussian white noise as the system input, and construct the vocal system. Finally, use the same evaluation criteria as sub model 1 to test the voacl system. Due to space constraints, this paper cannot display the selection results of the dimension of the basis function of all signals, but only the mean and standard deviation of the dimension of the three basis functions of all signals under the five model orders, which are recorded as $E_{q_{il}}$ and $D_{q_{il}}$ respectively, and their expressions are as follows:

35 $$\begin{aligned} & {E_{q_{il}}} = \frac{1}{{{N_b}}}\sum \limits _{k = 1}^{{N_e}} {{q_{il}}\left( k \right) } \end{aligned}$$

36 $$\begin{aligned} & {D_{q_{il}}} = \sqrt{ \frac{1}{{{N_b}}}\sum \limits _{k = 1}^{{N_e}} {{{\left( {{q_{il}}\left( k \right) - {E_{q_{il}}}} \right) }^2}} } \end{aligned}$$

where, $q_{il}(k)$ is the dimension corresponding to the *l*-*th* basis function $f_{lj}(n)$ of the *k*-*th* signal when the model order is *i*-*th*, and $N_b$ is the number of signals.

Table 2 shows the $E_{q_{il}}$ and $D_{q_{il}}$ of the signal collected in this paper under five model orders.

**Table 2:** $E_{q_{il}}/D_{q_{il}}$ of the signal collected in this paper

Model Order	$\textbf{4}$-$\textbf{th}$	$\textbf{6}$-$\textbf{th}$	$\textbf{8}$-$\textbf{th}$	$\textbf{10}$-$\textbf{th}$	$\textbf{12}$-$\textbf{th}$
$f_{1j}(n)$	15.72/2.37	16.03/2.13	16.12/2.05	16.24/1.89	16.30/1.90
$f_{2j}(n)$	15.79/2.36	16.14/2.16	16.20/2.16	16.47/2.07	16.55/2.03
$f_{3j}(n)$	15.53/2.27	15.83/2.12	15.75/2.08	15.83/1.95	15.94/1.91

After the dimension of the basis function is selected, TV-OPS is used to analyze the order of the model to determine whether the set order is redundant. If it is redundant, the redundant items are removed, and the weights of their respective regression coefficients and vectors are calculated. Limited by space, only the mean and standard deviation of TV-OPS results of all signals with model order of 6 are shown here, which are recorded as $E_{Cm}$ and $D_{Cm}$, and the expressions are as follows:

37 $$\begin{aligned} & E_{Cm}=\frac{1}{{{N_b}}}\sum \limits _{k = 1}^{{N_b}} {{C_{m6}\left( k \right) }} \end{aligned}$$

38 $$\begin{aligned} & D_{Cm}=\sqrt{ \frac{1}{{{N_b}}}\sum \limits _{k = 1}^{{N_b}} {{{\left( {{C_{m6}}\left( k \right) - {E_{Cm}}} \right) }^2}} } \end{aligned}$$

where, $C_{mi}(k)$ represents the *m*-*th* autoregressive coefficient and vector weight of the *k*-*th* signal when the model order is 6.

**Fig. 8:**
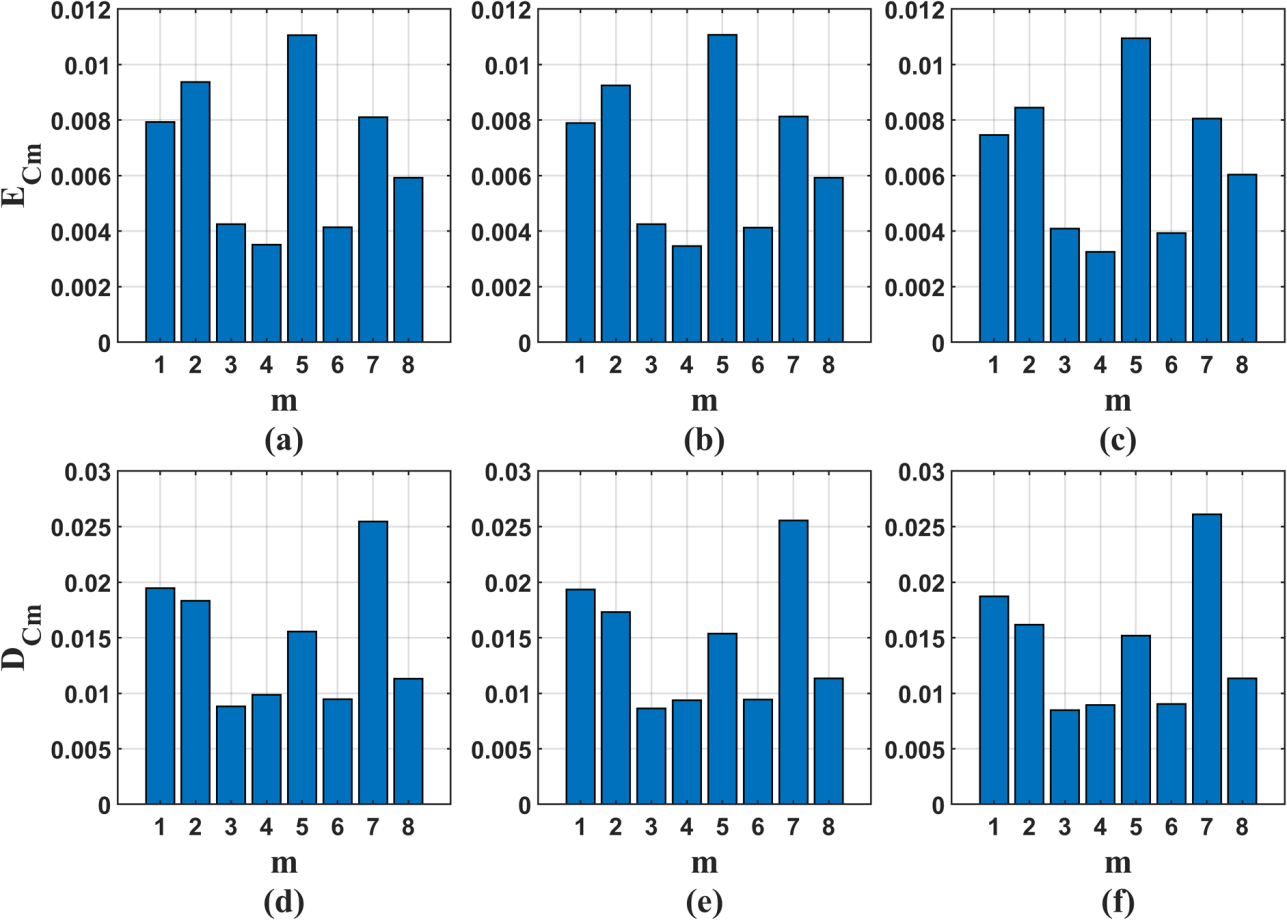
The $E_{Cm}$ and $D_{Cm}$ of TV-OPS results corresponding to three basis functions under 6-*th* order. **a** The $E_{Cm}$ corresponding to Legendre polynomial basis function; **b** The $E_{Cm}$ corresponding to Chebyshev basis function; **c** The $E_{Cm}$ corresponding to discrete cosine basis function; **d** The $D_{Cm}$ corresponding to Legendre polynomial basis function; **e** The $D_{Cm}$ corresponding to Chebyshev basis function; **f** The $D_{Cm}$ corresponding to discrete cosine basis function

Figure 8 shows the $E_{Cm}$ and $D_{Cm}$ of TV-OPS results corresponding to three basis functions under 6-*th* order.

**Fig. 9:**
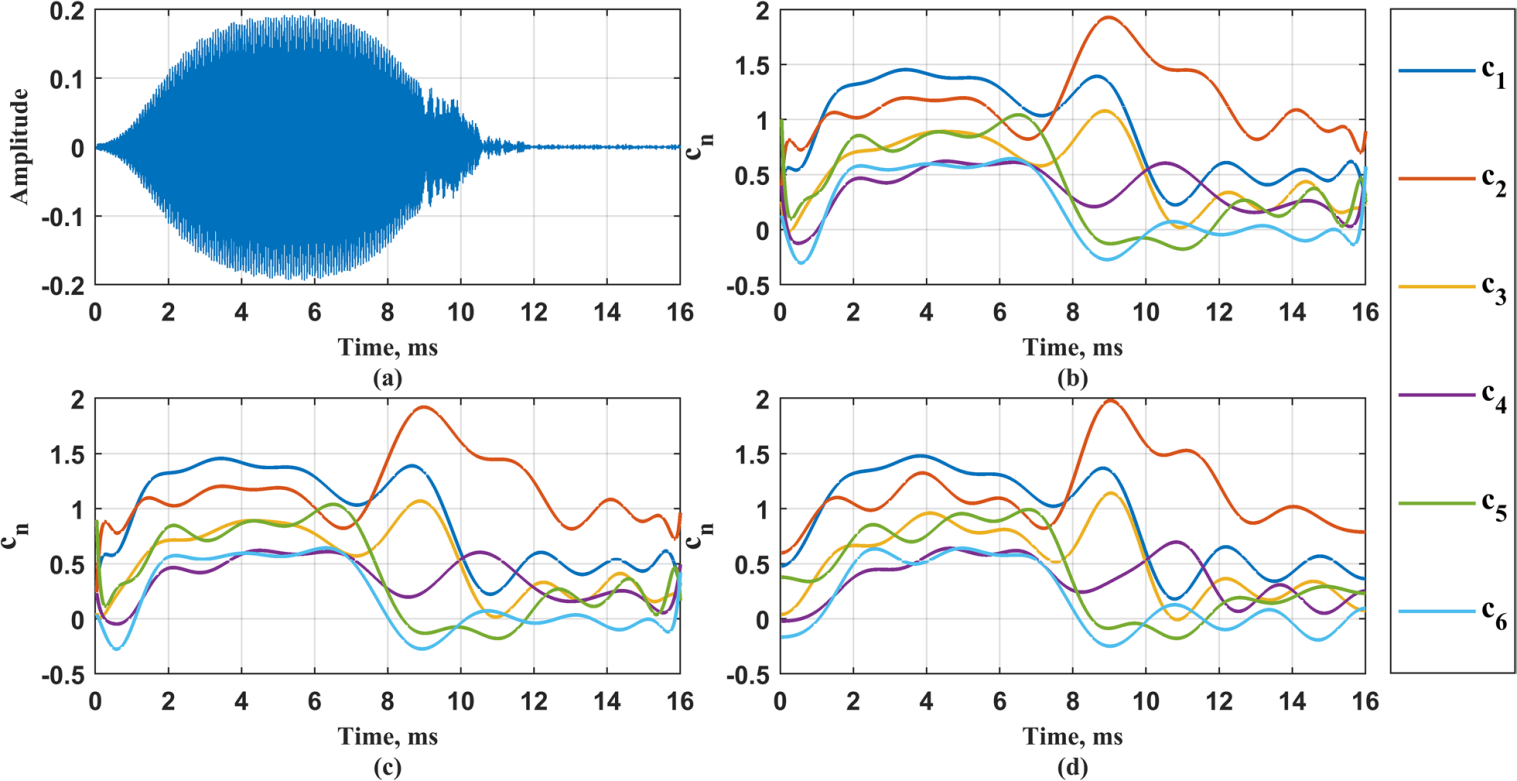
The $\textbf{c}_n$ of an echolocation signal obtained by three kinds of basis functions under the model order of 6. **a** Time domain diagram of the echolocation signal; **b** $\textbf{c}_n$ corresponding to Legendre polynomial basis function; **c** $\textbf{c}_n$ corresponding to Chebyshev basis function; **d** $\textbf{c}_n$ corresponding to discrete cosine basis function

**Fig. 10:**
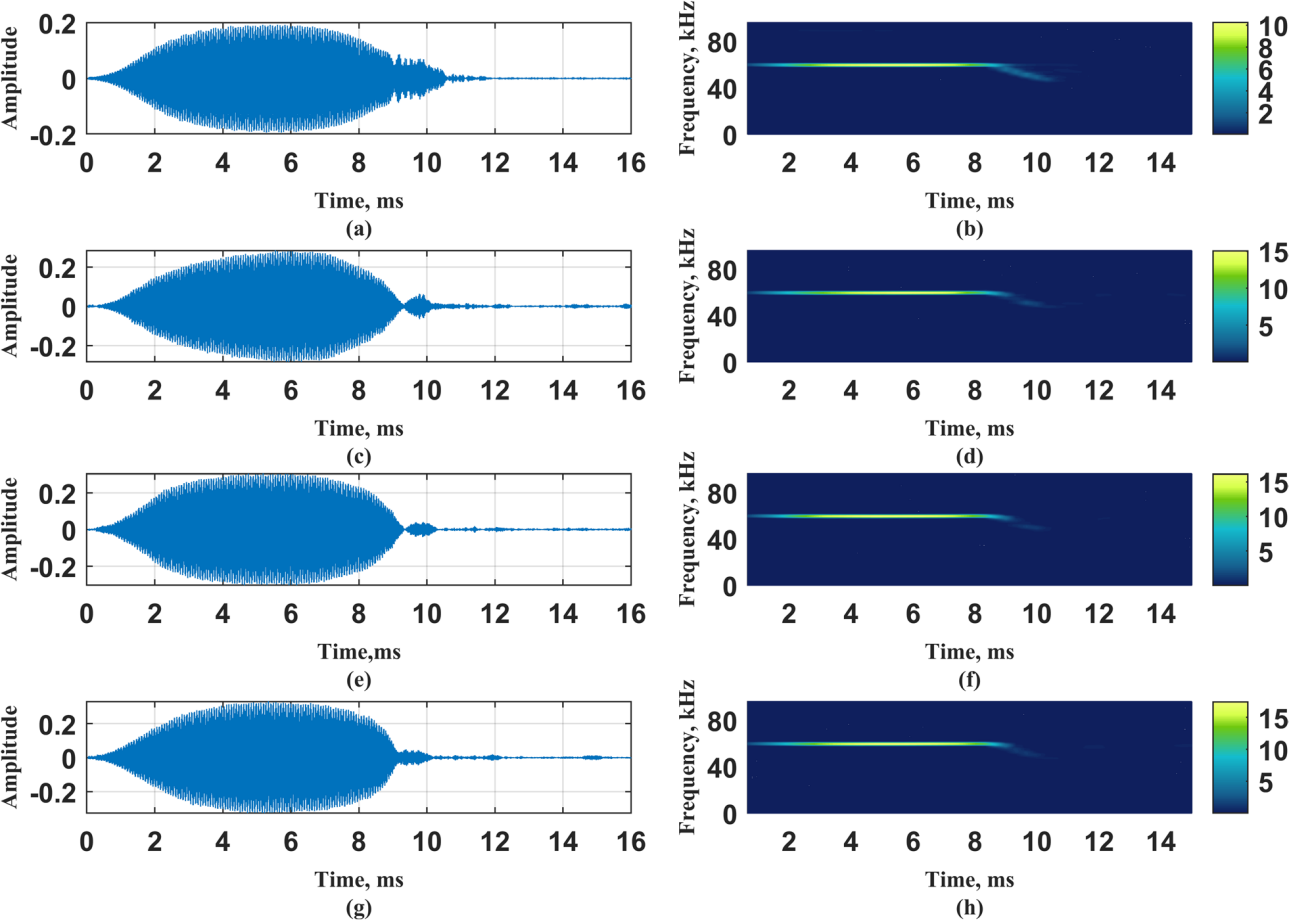
Time domain plot and STFT results of output signal and original signal. **a**Time domain diagram of original signal; **b** Original signal STFT results; **c** Time domain diagram of the output signal corresponding to the Legendre polynomial basis function; **d** The STFT result of the output signal corresponding to the Legendre polynomial basis function; **e** Time domain diagram of the output signal corresponding to the Chebyshev basis function; **f** The STFT result of the output signal corresponding to the Chebyshev basis function; **g** Time domain diagram of the output signal corresponding to the discrete cosine basis function; **h** The STFT result of the output signal corresponding to the discrete cosine basis function

After removing the redundant items through TV-OPS, the obtained time-varying autoregressive coefficient is constructed as a vocal system, the Gaussian white noise is used as the system input, and the obtained output signal is recorded as ${\hat{x}}_{ij}(n)$, the subscript *i* represents the order of the model, and *j* represents the selected basis function. Figure 9 shows the autoregressive coefficients of an echolocation signal obtained by three kinds of basis functions under the model order of 6-*th*. The Legendre polynomial basis function dimension is 18, the Chebyshev basis function dimension is 18, and the discrete cosine basis function dimension is 15. Figure 10 shows the output signals and STFT results of the vocal system corresponding to three different basis functions.

To assess the acoustic performance of sub model 2, the testing method is the same as that of sub model 1. For the basis function of a certain model order and a certain dimension, the obtained autoregressive coefficient is constructed as the vocal system of the corresponding order, and 5000 Monte Carlo tests are carried out to calculate the Pearson correlation coefficient between the single output signal and the original signal. The mean value of the Pearson correlation coefficient is taken as the linear correlation, which is recorded as $\rho _{ij}$, and its mathematical expression is as follows:

39 $$\begin{aligned} {\rho _{ij}}\left( k \right) = \frac{1}{{{N_m}}}\sum \limits _{m = 1}^{{N_m}} {\frac{{Cov\left( {{{\textbf{x}}_k},{{\hat{\textbf{x}}}^m}_{ij,k}} \right) }}{{{\sigma _{\textbf{x}}}{\sigma _{{{\hat{\textbf{x}}}^m}_{ij,k}}}}}} \end{aligned}$$

where, $N_m$ is the number of experiments, $\textbf{x}_k$ is the *k*-th echolocation signal, ${{\hat{\textbf{x}}}}_{ij,k}^m$ is the output signal obtained from the *m*-th experiment when the model order is *i* and the *j*-th basis function is selected as the basis function.

Calculate the linear correlation of all signals according to Eq. (39), and take the mean and standard deviation. The mean is recorded as $E_{ij}$, and the standard deviation is recorded as $D_{ij}$. The corresponding mathematical expression is as follows:

40 $$\begin{aligned} & {E_{ij}} = \frac{1}{{{N_e}}}\sum \limits _{k=1}^{{N_b}} {{\rho _{ij}}\left( k \right) } \end{aligned}$$

41 $$\begin{aligned} & {D_{ij}} =\sqrt{\frac{1}{{{N_e}}}\sum \limits _{k = 1}^{{N_b}} {{{\left[ {{\rho _{ij}}\left( k \right) - {E_{ij}}} \right] }^2}}} \end{aligned}$$

Table 3 shows the $E_{ij}$ and $D_{ij}$ corresponding to the three basis functions of the bat vocal system model constructed based on the signals collected in this paper under five model orders.

**Table 3:** $E_{ij}/D_{ij}$ of the signal collected in this paper

Model order	$\textbf{4}$-$\textbf{th}$	$\textbf{6}$-$\textbf{th}$	$\textbf{8}$-$\textbf{th}$	$\textbf{10}$-$\textbf{th}$	$\textbf{12}$-$\textbf{th}$
$f_{1j}(n)$	0.570/0.011	0.692/0.010	0.787/0.008	0.825/0.007	0.829/0.006
$f_{2j}(n)$	0.571/0.012	0.692/0.010	0.772/0.008	0.802/0.006	0.802/0.006
$f_{3j}(n)$	0.595/0.012	0.723/0.011	0.807/0.008	0.833/0.007	0.850/0.007

### Result analysis

From Table 1, it can be seen that reducing the adjustment coefficient $\lambda$ can improve the similarity mean, but this leads to an increase in the computational complexity of the iterative solution process, and the improvement obtained is too small, for example, the final results of $10\%$ and $15\%$ differ by only $1-2\%$ at each order. By comparing Tables 1 and  3, it can be seen that under the five model orders, the correlation mean $E_{ij}$ of sub model 2 line is higher than that of sub model 1, and the maximum similarity mean of sub model 2 is 0.85, which is higher than that of sub model 1 by 0.607, which verifies the conjecture of sub model 2 mentioned above, that is, in the process of bat vocalization, its vocal organs continue to function, and the corresponding vocal system parameters change continuously with time. However, due to the superior final results of the coefficient continuous variation model compared to the segmented constant model, and the wider applicability of the coefficient continuous variation model, we did not further analyze the interval length, which may be related to the bat species used in this paper. We speculate that bats that emit CF signals may be more suitable for the segmented constant model.

**Fig. 11:**
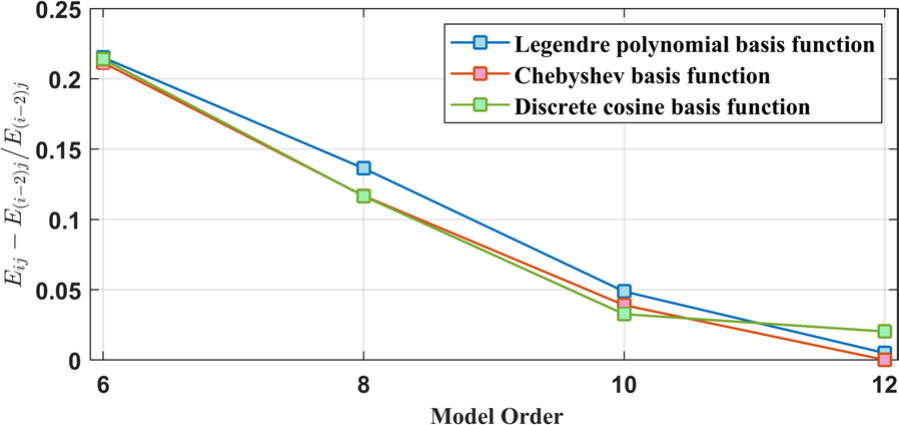
The growth rate of the correlation mean $E_{ij}$ of the three basis functions corresponding to the vocal system

According to Table 3, it can be found that with the increase of the order, the correlation mean $E_{ij}$ of the three basis functions corresponding to the vocal system increases, but the growth rate gradually slows down, as shown in Fig.  11, indicating that the correlation gain obtained by continuing to increase the order is getting smaller and smaller, and parameter redundancy may occur. In the vocal system corresponding to the three kinds of basis functions, the $E_{ij}$ of the discrete cosine basis function under the five model orders is higher than that of the other two basis functions, and the maximum value is 0.85, indicating that the output signal of the vocal system under the 12 order has $85\%$ similarity with the original signal, and the two have a high degree of matching, which can better characterize the bat vocal system. In addition, the correlation standard deviation $D_{ij}$ of the three basis functions corresponding to the vocal system shown in Table 3 is small under the five model orders, and decreases with the increase of the order, which shows that the vocal system corresponding to the three basis functions has good applicability.

## Discussion

As described in this paper, we propose a bat vocal system model based on the TV-AR model and construct two sub models, which are validated using 502 CF-FM bat echolocation signals. The results showed that the average linear similarity between the output signal of the bat vocalization system model (sub model 2) based on the coefficient continuous variation autoregressive model and the original signal reached $85\%$, indicating its high applicability. The advantage of modeling all vocal organs as a whole is that it can directly obtain the vocal characteristic parameters of bats, which can be used for subsequent identification of bat individuals of the same species and conducting bat colony experiments. In addition, this method is not limited to CF-FM bats and can probably be extended to FM bats (Appendix A). We have conducted preliminary analysis of FM echolocation signals using publicly available field data. As this is a preliminary technical validation, a more comprehensive evaluation will need to be conducted after our experimental conditions are met. The results indicate that compared to sub model 1, sub model 2 has higher applicability, although its average signal similarity is less than $85\%$ of Table  3, which is $73.4\%$ (Table 5). We believe this is due to uncertainties in field recording, such as environmental noise, and the need to redesign the basis functions of sub model 2 to better adapt to FM signals. Further analysis and experiments will be conducted after we capture FM bats and complete training.

However, since we model all vocal organs as a whole and use system parameters to characterize the overall changes of all organs during the vocal process, there may be parameter coupling problems, which makes it difficult to analyze individual vocal organs. Further subsystem models need to be established for individual organs and linked to their biological characteristics. Meanwhile, although the piecewise constant model may not perform as well as the coefficient continuous variation model in overall performance, its modeling effect on a certain vocal organ may be better than that of the coefficient continuous variation model. Therefore, the overall vocal system model obtained by combining multiple subsystem models will have higher applicability.

## Conclusions

Based on the vocal mechanism of bat and combined with TV-AR model, this paper proposes an equivalent model of bat vocal system with Gaussian white noise as input and echolocation signal as output. All vocal organs are regarded as a whole system, and the TV-AR model is used to describe the whole. Two guesses are put forward about the change trajectory of model parameters. Based on this conjecture, two sub models are proposed, which are based on piecewise constant coefficient autoregressive model and based on continuous coefficient autoregressive model. Parameters are solved by regularized least squares method and basis function method, traditional order criteria and TV-OPS are used for order analysis and redundancy removal, and the mean and standard deviation of the linear correlation degree between the output signal and the original signal are used as model performance evaluation criteria. The modeling results based on the measured signals of bats show that the output signal of the bat vocal system model based on the autoregressive model with continuously varying coefficients has $85\%$ similarity to the measured signal, which well simulates the bat vocal system. One point that needs to be pointed out is that this paper only models the overall sound organ system of bats, which is suitable for the overall modeling of the sound system. Subsequently, corresponding subsystem models will be established for each organ to further analyze the bat sonar system.?

It should be pointed out that this paper only models the bat’s larynx and supralaryngeal as a whole, and the corresponding subsystem models will be established for each part of the larynx and supralaryngeal (such as vocal fold, oral cavity and nasal cavity) in order to further analyze the bat sonar system.

## Figures and Tables

**Fig. 12:**
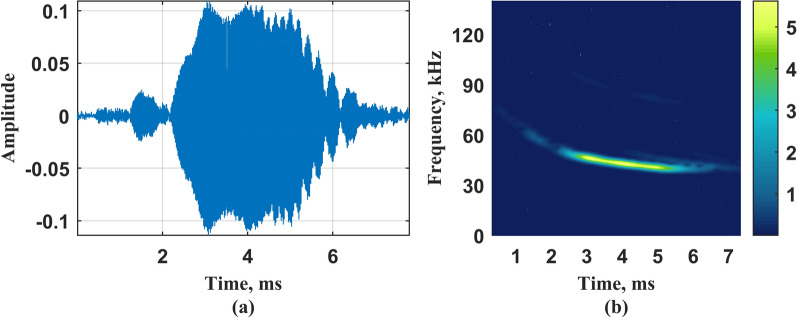
Time domain and time-frequency domain diagram of an FM signal. **a** Time domain diagram of an FM signal; **b** FM signal STFT results

**Fig. 13:**
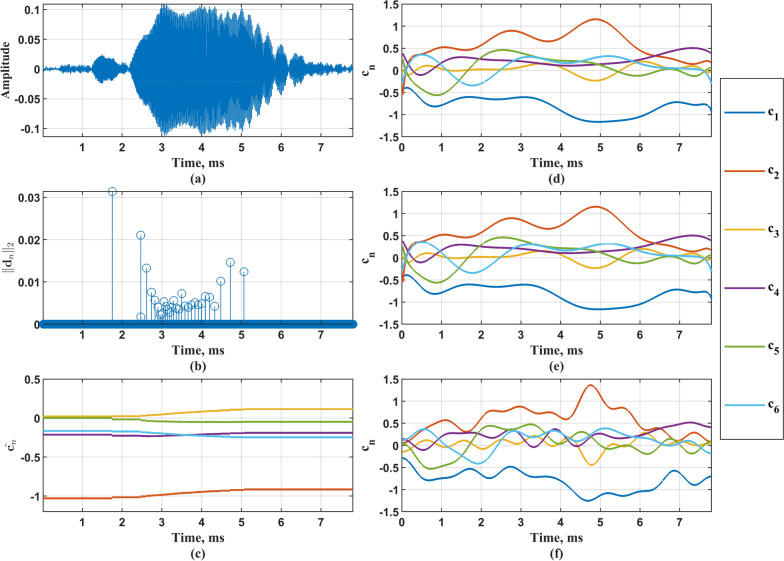
System parameters of sub model 1 ($5\%*{\lambda }^*$) and sub model 2 for FM signals at order 6-*th*. **a** Time domain diagram of an FM signal; **b** Change point sequence ${\left\| {{{\textbf{d}}_n}} \right\| _2}$; **c** Piecewise autoregressive constant coefficient ${\hat{\mathbf {{c}}}_n}$; **d** ${{\textbf{c}}_n}$ corresponding to Legendre polynomial basis function; **e** ${{\textbf{c}}_n}$ corresponding to Chebyshev basis function; **f** ${{\textbf{c}}_n}$ corresponding to discrete cosine basis function

## Data Availability

No datasets were generated or analysed during the current study.

## Preliminary evaluation of two sub models on FM bats

We conducted preliminary analysis using publicly available field data from the Sound library of the Museum National d’Histoire Naturelle, file number: MNHN-SO-2021-68, link: https://sonotheque.mnhn.fr/sounds/mnhn/so/2021–68 [43]. This data records the signals emitted by a male Epicoccus furinalis during field flight. We selected 56 signals with a sampling frequency of 384 kHz. Figure 12 shows the time-domain and time-frequency domain plots of one of the signals.

According to the two sub models described in this paper, the mean $E_{\text {G}\text {L},j}$ and standard deviation $D_{\text {G}\text {L},j}$ of signal similarity of FM signal on sub model 1 are shown in the Table 4.

**Table 4:** $E_{\text {G}\text {L},j}$ and ${D}_{\text {G}\text {L},j}$ of the FM signals under four adjustment coefficients and five orders

Model Order(xx$\%*\lambda ^{*}$)	$\textbf{4}$-$\textbf{th}$	$\textbf{6}$-$\textbf{th}$	$\textbf{8}$-$\textbf{th}$	$\textbf{10}$-$\textbf{th}$	$\textbf{12}$-$\textbf{th}$
$E_{\text {G}\text {L},j}/{D}_{\text {G}\text {L},j}\left( {5\% } \right)$	0.402/0.018	0.407/0.018	0.412/0.017	0.416/0.018	0.407/0.019
$E_{\text {G}\text {L},j}/{D}_{\text {G}\text {L},j}\left( {10\% } \right)$	0.361/0.016	0.356/0.017	0.371/0.018	0.378/0.019	0.380/0.019
$E_{\text {G}\text {L},j}/{D}_{\text {G}\text {L},j}\left( {15\% } \right)$	0.342/0.017	0.334/0.017	0.350/0.018	0.376/0.019	0.379/0.019
$E_{\text {G}\text {L},j}/{D}_{\text {G}\text {L},j}\left( {20\% } \right)$	0.321/0.017	0.329/0.018	0.331/0.018	0.338/0.019	0.341/0.018

The mean $E_{\text {G}\text {L},j}$ and standard deviation $D_{\text {G}\text {L},j}$ of signal similarity corresponding to sub model 2 are shown in the Table 5.

**Table 5:** $E_{ij}/D_{ij}$ of the FM signal

Model order	$\textbf{4}$-$\textbf{th}$	$\textbf{6}$-$\textbf{th}$	$\textbf{8}$-$\textbf{th}$	$\textbf{10}$-$\textbf{th}$	$\textbf{12}$-$\textbf{th}$
$f_{1j}(n)$	0.601/0.045	0.671/0.061	0.689/0.052	0.695/0.069	0.709/0.062
$f_{2j}(n)$	0.605/0.057	0.673/0.053	0.692/0.054	0.693/0.057	0.698/0.052
$f_{3j}(n)$	0.624/0.043	0.697/0.067	0.717/0.067	0.725/0.052	0.734/0.066

From the above two tables, it can be seen that sub model 2 performs better than sub model 1, corresponding to the time-varying autoregressive coefficients of the two sub models, which can be visually distinguished. Figure 13 shows the sequence of change points and corresponding piecewise constant autoregressive coefficients of sub model 1, as well as the time-varying autoregressive coefficients of sub model 2 (three basis functions).

From Fig. 13, it can be seen that the change point sequence of sub model 1 appears relatively concentrated during a certain period of time in the middle, and the time interval between adjacent change points is short. Within the same time period, the autoregressive coefficient of sub model 2 also shows significant changes. However, there is a large difference in the change trajectory between ${\hat{\mathbf {{c}}}_n}$ and ${{\textbf{c}}_n}$ during this period, and ${{\textbf{c}}_n}$ can better track the system parameter change trajectory, ultimately resulting in a higher average signal similarity of sub model 2 than sub model 1.
